# Inhibition of PKR by Viruses

**DOI:** 10.3389/fmicb.2021.757238

**Published:** 2021-10-25

**Authors:** Teresa Cesaro, Thomas Michiels

**Affiliations:** de Duve Institute, Université catholique de Louvain, Brussels, Belgium

**Keywords:** innate immunity, integrated stress response, mRNA translation, innate immunity evasion, viral proteins, double-stranded RNA

## Abstract

Cells respond to viral infections through sensors that detect non-self-molecules, and through effectors, which can have direct antiviral activities or adapt cell physiology to limit viral infection and propagation. Eukaryotic translation initiation factor 2 alpha kinase 2, better known as PKR, acts as both a sensor and an effector in the response to viral infections. After sensing double-stranded RNA molecules in infected cells, PKR self-activates and majorly exerts its antiviral function by blocking the translation machinery and inducing apoptosis. The antiviral potency of PKR is emphasized by the number of strategies developed by viruses to antagonize the PKR pathway. In this review, we present an update on the diversity of such strategies, which range from preventing double-stranded RNA recognition upstream from PKR activation, to activating eIF2B downstream from PKR targets.

## Introduction

### PKR: A Cornerstone in the Integrated Stress Response

The integrated stress response (ISR) is a signaling pathway that optimizes the cellular response to stress and aims to restore homeostasis after different types of stress (Pakos-Zebrucka et al., 2016). It relies on the detection of cellular stresses by 4 protein kinases, which are referred to as eIF2α kinases (EIF2AK) because they phosphorylate a common target: eukaryotic translation initiation factor 2 subunit alpha (EIF2S1 or eIF2α). eIF2α is a subunit of eIF2, which contributes to the formation of the ternary mRNA translation initiation complex. Phosphorylation of eIF2α Ser51 by eIF2α kinases tightens the interaction between eIF2α and eIF2B, a guanine exchange factor for eIF2, thereby preventing recycling of GDP-bound eIF2α and thus blocking translation initiation (Sudhakar et al., 2000). Translation blockade results in the rapid formation of stress granules (SGs; Anderson and Kedersha, 2008; McCormick and Khaperskyy, 2017).

While EIF2AK1 (HRI) is mostly sensing oxidative stress, EIF2AK3 (PERK) endoplasmic reticulum stress, and EIF2AK4 (GCN2) amino acid deprivation, EIF2AK2, better known as PKR, is an interferon-induced protein kinase activated *in primis* by viral double-stranded (ds) RNA molecules (Taniuchi et al., 2016). PKR was identified nearly 50years ago by the groups of D.H. Metz (Friedman et al., 1972) and I. Kerr (Kerr et al., 1977). In the 90s, human PKR cDNA was cloned at the Pasteur Institute (Meurs et al., 1990), opening the way to detailed molecular analysis of the PKR activation pathway and of the diversity of PKR activities.

PKR is a 551 amino acid-long protein, containing a C-terminal kinase domain and two N-terminal double-stranded RNA-binding motifs (DRBMs). It is mostly cytoplasmic although some PKR has been detected in the nuclear fraction (Jeffrey et al., 1995; Garcia et al., 2006). It is noteworthy that PKR as well as other proteins involved in innate antiviral immunity can be incorporated in stress granules together with translation initiation factors, SG-forming proteins and mRNA (Langereis et al., 2013; Onomoto et al., 2014). Stress granules are regarded as platforms required for innate immunity initiation and for activation of PKR itself (Onomoto et al., 2014; Reineke et al., 2015). Prolonged PKR activation can promote cell apoptosis. Both inhibition of viral mRNA translation and apoptosis of infected cells are effector mechanisms that critically limit viral spread in an infected host (Garcia et al., 2007).

PKR is also closely linked to p53. On the one hand, activated p53 upregulates the transcription of the gene coding for PKR, and PKR pro-apoptotic activity accounts for part of the tumor suppressor function of p53 (Yoon et al., 2009). On the other hand, PKR was shown to physically interact with p53 and to phosphorylate p53 *in vitro* (Cuddihy et al., 1999).

PKR further contributes to the inflammatory response by promoting NFκB activation through the activation of NIK and IKKB (Zamanian-Daryoush et al., 2000) and to the IFN response, by stabilizing IFN-β mRNA (Schulz et al., 2010).

PKR is thus a corner stone in the ISR as it links cellular stresses, such as DNA damage, to cell survival, innate immunity, and in particular antiviral response.

Given its critical role, PKR requires fine tuning. Excessive PKR activity can be detrimental, as is observed in Aicardi-Goutières syndrome patients where mutations in the adenosine deaminase 1 (ADAR1) lead to increased levels of endogenous dsRNA, thereby triggering PKR activation and uncontrolled IFN production (Chung et al., 2018).

### Triggers of PKR Activation

*EIF2AK2*, the gene encoding PKR, is constitutively expressed in mammalian cells. Its transcription can substantially be stimulated by IFN treatment because the promoter contains an IFN-stimulated response element (ISRE; Kuhen and Samuel, 1997). Splice variants have been described that affect exon 2 in the 5’UTR, which likely affect cell type-dependent translation (Kawakubo et al., 1999), or exon 7 in the coding region, which potentially generate a dominant-negative form of PKR (Li and Koromilas, 2001). The physiological impact of these variations however remains to be defined. Importantly, PKR is expressed as a latent enzyme, which requires further stimulation to become enzymatically active.

The best-characterized PKR activator is dsRNA, a typical by-product of RNA virus replication. Interestingly, dsRNA is also detectable by immunofluorescence in the cytoplasm of cells infected with DNA viruses, such as herpesviruses (Weber et al., 2006), where it was proposed to result from cytoplasmic relocalization of a pseudogene-encoded ribosomal RNA (Chiang et al., 2018). DsRNA can also be of endogenous origin, stemming in human cells from the annealing of mitochondrial or Alu sequence-derived transcripts. In physiological conditions, the concentration of endogenous dsRNA molecules is normally limited under the PKR activation threshold thanks to the dsRNA destabilizing activity of adenosine deaminase RNA specific 1 (ADAR1; Toth et al., 2009; Li et al., 2010; Okonski and Samuel, 2013).

Recently, circular RNAs, which are generated in the cell by a back-splicing mechanism, were shown to be potent PKR inhibitors. Such circular RNAs have a high propensity to form short (16–26 pb-long), imperfect, intramolecular RNA duplexes that inhibit PKR activity (Liu et al., 2019). Interestingly, upon viral infection, such circular RNAs undergo rapid degradation by RNase L (for review see Drappier and Michiels, 2015; Gusho et al., 2020), thus restoring PKR activity (Liu et al., 2019).

Other interactors, including RNAs and proteins, were shown to regulate PKR activation. Non-coding RNA 886 (nc886) was first identified as an inhibitor of PKR activation by dsRNA (Lee et al., 2011; Jeon et al., 2012). nc886 RNA was however shown to act as a PKR activator in stimulated T lymphocytes (Golec et al., 2019).

Proteins were also shown to regulate PKR activation by direct protein–protein contact.

Protein activator of interferon (IFN)-induced protein kinase EIF2AK2 (PRKRA) most commonly referred to as PACT (RAX in the mouse) was described as a PKR activator. PACT and PKR can interact through direct protein–protein interaction, *via* their dsRNA-binding domains (Huang et al., 2002). Direct interaction with PACT is sufficient to promote PKR activation *in vitro* and in cells.

Interestingly, another dsRNA-binding protein, TRBP can interact with both PACT and PKR, thus creating a complex regulatory network (Park et al., 1994). Upon stress, phosphorylation of PACT favors the release of PACT from the TRBP-PACT complex, thereby increasing the interaction of PACT with PKR and the consequent PKR activation (Singh et al., 2011).

### PKR Autoactivation Cascade

In response to dsRNA molecules or to other activation signals, PKR undergoes an autoactivation process. In the inactivated state, DRBM2 and probably DRBM1 keep the protein in a closed conformation through interaction of DRBMs with the kinase domain (Robertson and Mathews, 1996; Nanduri et al., 2000). Binding of dsRNA molecules to DRBMs causes the release of these domains from the kinase domain and the consequent dimerization and autophosphorylation of the protein (Garcia et al., 2006). Phosphorylation of threonines 446 and 451, considered as a primary marker of PKR activation, is crucial for PKR-mediated recognition of substrates like eIF2α and the consequent inhibition of mRNA translation (Dey et al., 2005). Autophosphorylation of other PKR residues, such as Ser33 (Wang et al., 2017) or Ser6 (Cesaro et al., 2021), likely results in fine tuning of PKR activity through a network of positive and negative feedbacks.

## Mechanisms of PKR Inhibition by Viruses

As outlined above, PKR is a critical player of the antiviral response and, since it acts by inhibiting mRNA translation, triggering apoptosis, and amplifying the IFN response, PKR acts as a broad range viral antagonist, inhibiting the replication of both RNA and DNA viruses.

**Figure 1:**
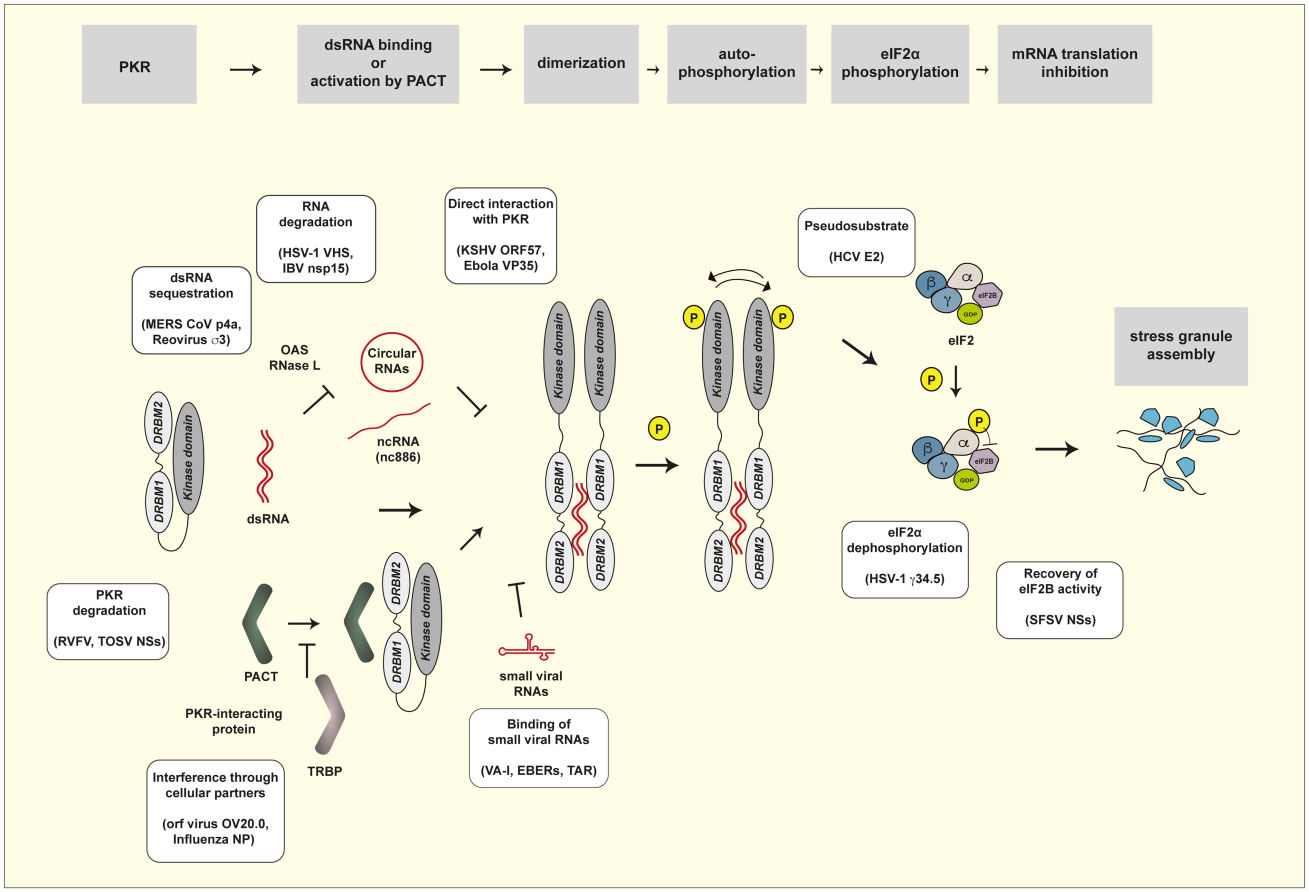
PKR activation pathway and viral countermeasures. Steps of the PKR activation pathway are framed in gray. Viral evasion mechanisms are presented in yellow frames. See Table 1 for a list of viral products involved in evasion of PKR activity.

As expected from the potent antiviral activity of PKR, many viruses evolved to counteract PKR activity by using their own viral products or by hijacking cellular proteins, acting at the different steps in the cascade of PKR activation (Figure 1). Previous reviews provide a broad view on the biology of PKR, its activation process, its many activities (Garcia et al., 2006, 2007), and its antiviral functions and viral countermeasures (Dauber and Wolff, 2009; Walsh and Mohr, 2011; Dabo and Meurs, 2012; Dzananovic et al., 2018).

This review provides an update on the diversity of mechanisms adopted by viruses to inhibit the PKR pathway, from upstream triggers to downstream targets.

Table 1 provides a list of viral products reported to be involved in evasion of the PKR response. The paragraphs below and Figure 1 review the different mechanisms by which these viral products counteract the PKR pathway.

**Table 1:** Strategies developed by viruses to escape PKR-mediated antiviral response.

Viral genome	Family	Virus	Viral product	Mechanism (ref)
ssRNA (+)	*Picornaviridae*	Theiler’s murine encephalomyelitis virus	L	Leader protein: very short protein processed from the N-terminal end of the polyprotein, rendering PKR “insensitive” to dsRNA (Borghese et al., 2019)
		Foot and mouth disease virus	3C	Viral protease responsible for viral polyprotein processing. Triggers PKR degradation (Li et al., 2017)
		Enterovirus A71	2A	Protease responsible for the primary cleavage of the viral polyprotein. Triggers the formation of atypical stress granules (Yang et al., 2018)
		Poliovirus	2A	
		Coxsackievirus A	2A	
	*Flaviviridae*	Japanese encephalitis virus	NS2A	Interaction with PKR, PKR dimerization inhibition (Tu et al., 2012)
		Dengue virus	NS4A	Recruitment of eIF4I to bypass PKR inhibition (Chen et al., 2015)
		Hepatitis C virus	NS5A	Interaction with PKR through formation of a complex involving cyclophilin A. Inhibits PKR dimerization (He et al., 2001; Sudha et al., 2012; Dabo et al., 2017; Colpitts et al., 2020)
			E2	Envelope protein. Acts as a PKR pseudosubstrate (Taylor et al., 1999)
	*Coronaviridae*	Infectious bronchitis virus	nsp2	PKR autophosphorylation inhibition and induction of eIF2α dephosphorylation by PP1-GADD34 (Wang et al., 2009)
			nsp15	Endonuclease. Acts by triggering RNA degradation (Gao et al., 2021; Zhao et al., 2021)
			?	Upregulation of GADD34, a subunit guiding PP1 to dephosphorylate eIF2α (Wang et al., 2009)
		Middle east respiratory syndrome virus	p4a	Accessory dsRNA-binding protein. Inhibits PKR when expressed from an heterologous virus (Rabouw et al., 2016)
ssRNA (−)	*Orthomyxoviridae*	Influenza virus A	NP	Nucleoprotein. Interaction with HSP40 and release of P58IPK (Polyak et al., 1996; Melville et al., 1999; Sharma et al., 2011)
		Influenza virus A and B	NS1	Direct interaction with PKR, binding to dsRNA (Dauber et al., 2006)
	*Paramyxoviridae*	Respiratory syncytial virus	N	Nucleoprotein. PKR sequestration and induction of PP2 phosphatase (Groskreutz et al., 2010)
		Human parainfuenza virus 3	NP	Nucleoprotein. Inhibition of stress granules by shielding of viral mRNAs (Hu et al., 2018)
	*Filoviridae*	Ebola virus, Marburg virus	VP35	dsRNA-binding protein acting as a co-factor for the polymerase complex. Binds dsRNA, PACT and PKR, the latter activity being the most effective one (Schumann et al., 2009; Hume and Mühlberger, 2018)
	*Bunyaviridae*	Hantavirus	NP	Nucleoprotein. PKR dimerization inhibition (Wang and Mir, 2015)
		Rift valley fever virus	NSs	Proteasomal degradation of PKR (Kalveram et al., 2013; Mudhasani et al., 2016)
		Toscana virus	NSs	Proteasomal degradation of PKR (Ikegami et al., 2009; Kalveram and Ikegami, 2013)
		Sicilian phlebovirus	NSs	rescue of eIF2B guanine nucleotide exchange activity (Wuerth et al., 2020)
dsRNA	*Reoviridae*	Avian reovirus	p17	PKR-dependent autophagy induction (Chi et al., 2019)
			σA	PKR autophosphorylation inhibition (Gonzalez-Lopez et al., 2003)
		Mammalian reovirus	σ3	Outer capsid protein. dsRNA-binding protein responsible for a strain-dependent local PKR inhibition. PKR inhibition is partly independent of dsRNA binding (Schmechel et al., 1997; Jacobs and Langland, 1998; Smith et al., 2005; Guo et al., 2021)
RNA/DNA	*Retroviridae*	Human immunodeficiency virus 1	Tat	Transcriptional activator acting by binding the TAR RNA sequence. Acts by direct interaction with PKR and as a PKR pseudosubstrate (McMillan et al., 1995; Brand et al., 1997)
			TAR	RNA sequence formed by the HIV transcript. Binds PKR and inhibits PKR dimerization (Gunnery et al., 1990; Heinicke et al., 2009; Sunita et al., 2015). Interacts with TRBP (Sanghvi and Steel, 2011)
dsDNA	*Adenoviridae*	Adenovirus	VAI RNA	Short RNAs abundantly expressed in infected cells. Interact with PKR, thereby preventing PKR dimerization and autophosphorylation (Price and Penman, 1972; Mathews and Shenk, 1991; Sharp et al., 1993; Launer-Felty et al., 2015; Dzananovic et al., 2017; Hood et al., 2019); for review see (Punga et al., 2020).
			E1B-55k	PKR autophosphorylation inhibition (Spurgeon and Ornelles, 2009)
			E4orf6	PKR autophosphorylation inhibition (Spurgeon and Ornelles, 2009)
		Mouse adenovirus 1	?	PKR degradation (Goodman et al., 2019)
	*Herpesviridae*	Herpes simplex 1 virus	ICP34.5	Acts as a PP1 regulatory subunit, leading PP1 to dephosphorylate eIF2α (He et al., 1998)
			Us11	Direct interaction with PKR, PKR autophosphorylation inhibition, PKR pseudosubstrate, (PACT interaction; Poppers et al., 2000; Cassady and Gross, 2002; Peters et al., 2002)
			VHS	Tegument nuclease triggering RNA degradation (Dauber et al., 2011)
		Epstein–Barr virus	SM	Direct interaction with PKR, binding to dsRNA, PKR autophosphorylation inhibition (Poppers et al., 2003)
			EBER1 and 2	Short RNAs abundantly expressed in infected cells. Interact with PKR, thereby preventing PKR dimerization and autophosphorylation (Greifenegger et al., 1998; Nanbo et al., 2002; McKenna et al., 2007)
		Kaposi’s sarcoma-associated herpesvirus	LANA2	Protein expressed during latency. Inhibits eIF2α phosphorylation (Esteban et al., 2003)
			ORF57	Nuclear protein involved in maturation and stability of viral mRNAs. Inhibits PKR through direct interaction with PKR dsRNA-binding motifs (Sharma et al., 2017)
		Cytomegalovirus	IRS1	dsRNA-binding protein that is non-essential but involved in viral replication. Inhibits PKR, through dsRNA or direct PKR binding (Marshall et al., 2009)
			TRS1	dsRNA-binding protein that is non-essential but involved in viral replication. Inhibits PKR, through dsRNA or direct PKR binding (Marshall et al., 2009)
		Mouse cytomegalovirus	m142, m143	dsRNA-binding proteins preventing PKR autophosphorylation (Valchanova et al., 2006)
	*Poxviridae*	Vaccinia virus	K3L	PKR autophosphorylation inhibition, PKR pseudosubstrate (Davies et al., 1992, 1993; Carroll et al., 1993)
			E3L	dsRNA sequester, direct interaction with PKR (Davies et al., 1993; Beattie et al., 1995; Romano et al., 1998)
			K1L	Cytoplasmic protein required for productive virus infection. Triggers a reduction of dsRNA amounts (Willis et al., 2011)
		Orf virus	OV20.0	dsRNA-binding protein, acting through interaction with PKR and PACT (Tseng et al., 2015; Liao et al., 2021)

### dsRNA Sequestration, Masking, or Degradation

A key mechanism used by viral proteins to inhibit PKR-mediated antiviral response is hiding or sequestering dsRNA molecules that would otherwise activate PKR. An example of such a dsRNA sequestering viral proteins is Middle East respiratory coronavirus (MERS-CoV) protein 4a (Rabouw et al., 2016). Historical examples of viral dsRNA-binding proteins include the E3L protein of vaccinia virus (VACV; Romano et al., 1998), the NS1 protein of Influenza virus (Dauber et al., 2006), and the σ3 outer capsid protein of mammalian reovirus that was shown to compete with PKR for dsRNA binding *via* its C-terminal DRBM (Jacobs and Langland, 1998). For the latter three proteins however, PKR inhibition was later shown to rely on their ability to form direct protein–protein contacts with PKR (Davies et al., 1993; Guo et al., 2021), sometimes in a strain-dependent manner (Min et al., 2007).

Interestingly, some viruses evolved to restrict PKR activation by limiting dsRNA availability through degradation. This was shown for the nsp15 endonuclease of Infectious Bronchitis Virus (IBV), an avian coronavirus (Gao et al., 2021; Zhao et al., 2021), and for the virion host shutoff (VHS) tegument protein, a ribonuclease encoded by herpes simplex 1 (Dauber et al., 2011).

In the case of Human parainfluenza virus type 3 (HPIV3), a negative-stranded RNA virus, association of nucleo (N) and phospho (P) proteins is responsible for the formation of inclusion bodies, which shield newly synthesized viral RNA, thereby inhibiting PKR activation and formation of stress granules. In this case however, mRNA, which is shielded by inclusion bodies, rather than dsRNA, is likely to be the trigger of PKR activation (Hu et al., 2018).

### PKR Degradation

Toscana virus (TOSV) and Rift valley fever virus (RVFV) are two members of the *Bunyaviridae* family, which trigger proteasomal degradation of PKR through their non-structural NSs proteins (Ikegami et al., 2009; Kalveram et al., 2013). TOSV NSs was shown to interact with PKR but it is unclear how this interaction triggers proteasomal degradation of PKR (Kalveram and Ikegami, 2013). In the case of RVFV, NSs carries out this activity by binding to PKR and to F-box and WD repeat domain containing 11 (FBXW11), thus assembling an E3 ubiquitin ligase complex, which triggers PKR polyubiquitination and its consequent degradation by the proteasome (Mudhasani et al., 2016).

Adenovirus late viral proteins E1B-55k and E4orf6 are both multifunctional proteins that can block p53-dependent apoptosis, interfere with mRNA export from the nucleus, and regulate viral replication. In addition, these proteins are involved in the formation of E3 ubiquitin-protein ligase complex with cullin 5, Ring-box 1, and elongins B and C. E1B-55k and E4orf6 inhibit PKR and eIF2α phosphorylation at late stages of infection in a cullin 5-dependent manner, suggesting that these proteins may act by triggering PKR degradation. However, their PKR antagonist activity may also depend on their influence on subcellular RNA trafficking (Spurgeon and Ornelles, 2009). Proteasome-dependent degradation is, however, more likely as this mechanism was recently documented in the case of the mouse adenovirus type 1 (Goodman et al., 2019).

A typical way used by picornaviruses and other positive-stranded RNA viruses to escape immunity is to cleave immune sensor and effector proteins with proteases that are encoded by these viruses to process their own polyprotein. In the case of enteroviruses, such as poliovirus, coxsackievirus, or enterovirus A-71, a recent high-throughput study identified hundreds of host proteins that are substrates of 2A or 3C proteases. PKR was surprisingly not in the list (Saeed et al., 2020). In contrast, 3C protease of another picornavirus, foot and mouth disease virus (FMDV), was shown to trigger PKR degradation. In this case however, PKR was not a direct substrate of protease 3C but PKR degradation occurred through the lysosomal pathway (Li et al., 2017).

### Inhibition of PKR Dimerization and Autophosphorylation

Many viral products were shown to inhibit PKR activation and autophosphorylation without evidence for direct interaction with dsRNA or with PKR itself. These viral products likely prevent dsRNA binding, dimerization, and/or autophosphorylation of PKR but the precise mechanism by which they act is not fully elucidated.

These include, for instance, m142 and m143 of the murine cytomegalovirus (MCM; Valchanova et al., 2006), or nsp2 of Infectious bronchitis virus (IBV; Wang et al., 2009). Hantavirus escapes PKR-mediated antiviral response by inhibiting PKR dimerization with its nucleoprotein (NP). However, competitive binding of NP to dsRNA or to PKR itself could not be documented (Wang and Mir, 2015).

### PKR Inhibition Through Direct Interaction

#### Interacting Viral RNAs

Inhibition of PKR by physical interaction not only involves viral proteins but also virus-encoded RNAs. This was well documented for Adenovirus, which produces a highly structured 160nt viral RNA called VA-I RNA, that interacts with PKR and inhibits its activation (Price and Penman, 1972; Mathews and Shenk, 1991; for review, see Punga et al., 2020). Similar short transcripts named EBERs (EBER-1 and EBER-2), transcribed lately during Epstein–Barr virus infection, were shown to bind to and inhibit PKR, thereby conferring resistance to IFN-induced apoptosis in Burkitt lymphoma cells (Greifenegger et al., 1998; Nanbo et al., 2002). EBERs and VA-I are extremely abundant viral transcripts. They were shown to compete for PKR binding and to bind PKR with high affinity (K_d_ *ca.* 0.3nM; Sharp et al., 1993).

Tat-responsive region RNA (TAR) of human immunodeficiency virus 1 (HIV-1) is another viral RNA that shares the capacity to inhibit PKR activity (Gunnery et al., 1990). Interestingly, TAR RNA forms a 23bp hairpin that binds PKR monomers but cannot accommodate PKR dimers because PKR dimer binding requires a dsRNA stretch longer than 30bp. TAR RNA can also self-associate, thus forming longer dsRNA molecules, which show the ability to activate PKR *in vitro* (Heinicke et al., 2009). Interestingly, increasing the concentration of dsRNA, even of long dsRNA species that have the capacity to activate PKR, leads to PKR inhibition, likely because PKR monomers are diluted out on separate dsRNA molecules and have therefore decreased ability to dimerize (Heinicke et al., 2009; Sunita et al., 2015).

VA-I RNA structure, examined by many biochemical approaches (see Punga et al., 2020), and more recently by small-angle X-ray scattering (SAXS; Launer-Felty et al., 2015) and X-ray crystallography (Hood et al., 2019), displays an elongated apical stem, a central domain, and a short terminal stem. The apical stem forms a highly stable 22bp helix allowing PKR binding and carrying several wobble nucleotide pairs, which surprisingly appear to tune down slightly the inhibitory activity of VA-I RNA (Hood et al., 2019). The central domain of VA-I, which contains a pseudoknot structure and a conserved tetranucleotide stem, is essential for PKR inhibition and presumably acts by preventing PKR dimerization (Launer-Felty et al., 2015; Dzananovic et al., 2017; Hood et al., 2019).

In conclusion, virus-encoded small RNAs appear to act by preventing PKR dimerization in two different ways: (i) through their abundance, they trap PKR monomers and decrease the chances of PKR dimerization on a single-dsRNA molecule; (ii) through their structure, they inhibit dimerization *via* a still-elusive mechanism.

#### Interacting Viral Proteins

Some viral proteins were shown to bind PKR through direct protein–protein interaction, thereby blocking PKR autophosphorylation, dimerization, or phosphorylation of eIF2α. Examples include the nucleoprotein (N) of Respiratory syncytial virus (RSV; Groskreutz et al., 2010), Tat from HIV-1 (McMillan et al., 1995; Brand et al., 1997), and ORF57 from Kaposi’s sarcoma-associated herpes virus (KSVH; Sharma et al., 2017). NS5A of hepatitis C was found to bind the dimerization domain of PKR in a two-hybrid screen and in transfected COS-1 cells (Gale et al., 1997, 1998). Although no evidence was provided that PKR is inhibited by NS5A during HCV infection (Dabo and Meurs, 2012), substituting NS5A for E3L in VACV showed PKR inhibition in infected cells (He et al., 2001). Some proteins inhibit PKR kinase activation by interacting with PKR as pseudosubstrates. Examples include the E2 envelope protein of hepatitis C virus (Taylor et al., 1999) and K3L of VACV (Davies et al., 1992).

Interestingly, a number of viral proteins were shown to bind both PKR and dsRNA. These include the NS1 protein of Influenza virus and the E3L protein of VACV referred to above, but also the Us11 protein from Herpes simplex 1 virus (HSV-1; Poppers et al., 2000; Cassady and Gross, 2002), the related early Sm protein of Epstein–Barr virus (EBV; Poppers et al., 2003) or the TRS1 protein produced by the human cytomegalovirus (CMV; Marshall et al., 2009). In the latter case, although TRS1 residues required for PKR and dsRNAs binding do not fully overlap, interaction with both substrates is required to achieve full PKR inhibition (Bierle et al., 2013). The VP35 protein encoded by filoviruses, such as Ebola and Marburg viruses, was also reported to interact with both dsRNA and PKR, through a C-terminal domain called IID. However, mutations in this domain that affect dsRNA binding do not affect PKR inhibition, suggesting that dsRNA binding by VP35 is not mandatory for PKR inhibition (Schumann et al., 2009).

### PKR Inhibition Through Cellular Interacting Proteins

As introduced above, several host proteins were reported to regulate PKR in either a positive or a negative fashion.

TRBP, a PKR inhibitor, was discovered as protein binding to the TAR RNA sequence of HIV (Gatignol et al., 1991). TAR can also bind to and activate PKR. In HIV-infected cells, however, TRBP was shown to contribute to PKR inhibition although the precise mechanism of this inhibition is unclear (Sanghvi and Steel, 2011).

PACT can be targeted as a PKR evasion strategy. In addition to binding to dsRNA and PKR itself (Liao et al., 2021), the Orf virus (ORFV)-encoded protein OV20.0 was shown to interact with PACT, thereby blocking PACT-mediated PKR activation (Tseng et al., 2015).

As referred to above, Us11 of HSV-1 uses its RNA-binding domain to interact with PKR kinase, leading to the prevention of eIF2α phosphorylation. Us11 was also shown to interact with PACT, suggesting an indirect mechanism of PKR inhibition as above. It was, however, shown that Us11 interaction with PKR was more important than interaction with PACT for Us11-mediated PKR inhibition (Peters et al., 2002). The situation is very similar in the case of filovirus VP35 proteins. In addition to binding dsRNA and PKR, Marburg virus VP35 also interacts with PACT. PKR inhibition does, however, not seem to rely on direct binding to PACT because PKR inhibition by VP35 turned out to be cell type-dependent and was not restored by ectopic expression of PACT (Hume and Mühlberger, 2018).

Influenza virus is able to induce PKR inhibition through activation of DnaJ heat shock protein family (Hsp40) member C3, known as P58^IPK^, which is one of the cellular PKR inhibitors (Lee et al., 1994). P58^IPK^ forms a complex with other heat shock proteins (Hsp) Hsp40 and Hsp70 where it is not active. The nucleoprotein (NP) from Influenza A virus can associate with HSP40, thereby leading to the dissociation of P58^IPK^ from the chaperone complex. Free P58^IPK^ in turn acts to inhibit PKR (Polyak et al., 1996; Melville et al., 1999; Sharma et al., 2011).

### eIF2α Dephosphorylation

Some viruses evolved to act downstream of the PKR pathway, by triggering the dephosphorylation of phospho-eIF2α.

IBV infection was shown to upregulate the transcription of GADD34, a co-factor of the PP1 phosphatase, which guides this phosphatase toward specific substrates including phospho-eIF2α, thereby preventing PKR-mediated translation inhibition (Wang et al., 2009).

Similarly, ICP34.5 protein of HSV-1 can substitute for GADD34 by complexing the PP1 phosphatase *via* its C-terminus and redirecting PP1 toward phospho-eIF2α (He et al., 1998).

In the context of RSV, the nucleoprotein (N) was found to recruit PP2, which in turn binds to eIF2α, causing its dephosphorylation and permitting viral spread (Groskreutz et al., 2010).

### Acting Downstream From eIF2α

Lately, it has been shown that another Bunyavirus, sandfly sicilian phlebovirus (SFSV), can indirectly escape the PKR response by acting on eIF2B, the guanine nucleotide exchange factor whose activity is prevented when bound to phospho-eIF2α. Data of Wuerth et al. suggest a model where the NSs protein of SFSV would bind the eIF2B-eIF2 complex (that includes eIF2α), thereby modifying the structure of the complex in such a way to restore eIF2B guanine nucleotide exchange activity despite eIF2α Ser51 phosphorylation (Wuerth et al., 2020).

NS4A protein from Dengue virus (DENV) has been shown to evade the innate immune response by a different mechanism. The protein can bind eIF4I and supports DENV replication in the cells. Knockdown of eIF4I surprisingly decreased PKR and eIF2α phosphorylation levels. This shows that the viral protein is able to limit PKR activation by sequestering a potential direct or indirect activator of PKR (Chen et al., 2015).

### Additional Mechanisms

*PKR desensitization*: Through a still undefined mechanism, the leader (L) protein of Theiler’s murine encephalomyelitis virus (TMEV) can act to prevent dsRNA recognition by PKR and inhibit stress granule formation although the L protein does not interact with dsRNA (Borghese and Michiels, 2011; Borghese et al., 2019).

*Activation of PKR*: in contrast to other viral proteins, p17 from ARV was shown to subvert the innate immune response by triggering PKR. In this case, activation of PKR contributed to triggering autophagy, which was found to increase virus replication (Chi et al., 2013, 2019). Other viruses take advantage of some extent of PKR activation. For instance, reoviruses which use the σ3 dsRNA-binding protein to dampen PKR activation still benefit from some level of PKR activation to trigger protein synthesis shutoff (Smith et al., 2005). Similarly, HCV, which was reported to inhibit PKR through proteins NS5A and E2, was proposed to take advantage of some level of PKR activation to inhibit IFN mRNA translation while IRES-mediated translation of its own genome was not affected by eIF2α phosphorylation (Arnaud et al., 2010; Kim et al., 2011).

## Discussion

### Acting Upstream or Downstream From the Pathway?

At first glance, it would look more effective for viral proteins to act upstream from PKR activation, by shielding dsRNA. Indeed, in addition to inhibiting PKR activation, such proteins are expected to prevent equally the activation of the other innate immune response pathways that depend on dsRNA recognition, such as the MDA5/MAVS pathway leading to IFN expression, or the oligoadenylate synthetase/RNaseL pathway leading to RNA degradation and IFN response amplification (Drappier and Michiels, 2015; Tan et al., 2018).

It is therefore unclear why some viruses evolved to act on downstream steps, for instance by triggering specific PKR degradation. It may be considered that a too broad inhibition of innate immunity would be detrimental to the virus because uncontrolled viral spread may lead to enhanced virus detection by the immune response or to premature death of the host, thus decreasing the chances of host-to-host transmission. Viruses possibly evolved to target specific arms of the innate immune response according to the cell type that they infect.

It is noteworthy that, acting at the other end of the pathway, downstream from eIF2α phosphorylation leads to other effects. Indeed, eIF2α phosphorylation is the convergence point of distinct arms of the ISR, involving the four eIF2α kinases: PKR, PERK, GCN2, and HRI (Taniuchi et al., 2016). Thus, viruses, such as IBV, which promote eIF2α dephosphorylation by hijacking cellular phosphatases (Wang et al., 2009) or viruses, such as SFSV, which prevent eIF2B inhibition (Wuerth et al., 2020) not only escape PKR but also PERK activity. Escaping PERK activity is likely important for such enveloped viruses, which may trigger endoplasmic reticulum stress due to massive viral glycoprotein exportation. Note that GCN2 and to a lesser extent HRI were also suggested to play antiviral roles and to be targeted by viruses (Liu et al., 2020).

### Targeting Multiple Steps of the Pathway

Some viruses devote more than one coding region of their genome to the inhibition of the PKR pathway.

K3L (Davies et al., 1992; Carroll et al., 1993) and E3L (Beattie et al., 1995; Romano et al., 1998) proteins from VACV both contribute to PKR phosphorylation inhibition: the former, by binding to PKR, acts as a PKR pseudosubstrate to inhibit phosphorylation of eIF2α, while the latter acts by interacting with both dsRNA and PKR to mediate the inhibition.

In the case of Infectious bronchitis virus (IBV), three mechanisms have been proposed to be involved in the inhibition of the PKR pathway. First, the nsp15 endonuclease encoded by this virus was proposed to trigger the degradation of PKR-activating RNA molecules in infected cells (Gao et al., 2021; Zhao et al., 2021). Next, infection by IBV was reported to lead to a transcriptional upregulation of the gene coding GADD34, thus enhancing PP1-mediated dephosphorylation of eIF2α (Wang et al., 2009). In the same work, it was shown that IBV Nsp2 displayed a weak PKR antagonist activity, although the mechanism of PKR inhibition by this protein was not elucidated (Wang et al., 2009). In this case, targeting multiple players in the PKR pathway can not only increase the potency of PKR inhibition but can also help to evade other innate immunity pathways.

### Future Prospects

More and more studies emphasize the possibility to regulate PKR activation through posttranslational modifications, such as SUMOylation, ISGylation, ubiquitination, and phosphorylation. As reported above, NSs proteins of bunyaviruses like RVFV can assemble an ubiquitin ligase complex, which targets PKR for proteasomal degradation (Mudhasani et al., 2016).

In contrast, although ISGylation and SUMOylation were shown to modulate PKR activity (Okumura et al., 2013; de la Cruz-Herrera et al., 2014; Maarifi et al., 2018), no viral protein has been identified yet that would trigger PKR posttranslational modification by the attachment of ISG15 or SUMO. It is likely that such proteins exist but remain to be identified.

Phosphorylation is another posttranslational modification involved in activation and fine tuning of PKR activity. Although viruses are well known to trigger extensive signal transduction cascades through phosphorylation by virus-encoded and cellular kinases, to the best of our knowledge, no viral PKR escape mechanism has been deciphered that would be based on inhibitory phosphorylation of PKR residues. The recent development of high-throughput phosphoproteomic methods might hopefully lead to new discoveries in this field.

Although this review focuses on the antiviral activity of the PKR-eIF2α axis and viral countermeasures, it is important to keep in mind that PKR activity is not limited to translation inhibition. PKR is also connected to other diverse and critical pathways, including mitosis and apoptosis control by p53, inflammation control through NFκB activation (Bennett et al., 2012), IFN production (Schulz et al., 2010), and even neuronal homeostasis (Gal-Ben-Ari et al., 2018). The involvement of PKR in these pathways suggests many alternative ways by which PKR might control viral infection and influence virus evolution.

## Author Contributions

TC wrote the first draft of the manuscript. TC and TM wrote sections of the manuscript. All authors contributed to the article and approved the submitted version.

## Funding

TC was the recipient of an Aspirant fellowship of the FNRS. Work was supported by the EOS joint programme of Fonds de la recherche scientifique-FNRS and Fonds wetenschapellijk onderzoek-Vlaanderen-FWO (EOS ID: 30981113) by national lotery players and Actions de Recherche concertée (ARC).

## Conflict of Interest

The authors declare that the research was conducted in the absence of any commercial or financial relationships that could be construed as a potential conflict of interest.

## Publisher’s Note

All claims expressed in this article are solely those of the authors and do not necessarily represent those of their affiliated organizations, or those of the publisher, the editors and the reviewers. Any product that may be evaluated in this article, or claim that may be made by its manufacturer, is not guaranteed or endorsed by the publisher.
